# Resource allocation optimization with multi-trait genomic prediction for bread wheat (*Triticum aestivum* L.) baking quality

**DOI:** 10.1007/s00122-018-3186-3

**Published:** 2018-09-19

**Authors:** Bettina Lado, Daniel Vázquez, Martin Quincke, Paula Silva, Ignacio Aguilar, Lucia Gutiérrez

**Affiliations:** 1 Department of Statistics, Facultad de Agronomía, Universidad de la República, Garzón 780, 12900 Montevideo, Uruguay; 2 Department of Agronomy, University of Wisconsin– Madison, 1575 Linden Dr, Madison, WI 53706 USA; 3 Instituto Nacional de Investigación Agropecuaria, Est. Exp. La Estanzuela, Ruta 50 km 11.5, 70006 Colonia, Uruguay; 4 Instituto Nacional de Investigación Agropecuaria, Est. Exp. Las Brujas, Ruta 48 km 10, Rincón del Colorado, 90200 Canelones, Uruguay

## Abstract

**Key Message:**

Multi-trait genomic prediction models are useful to allocate available resources in breeding programs by targeted phenotyping of correlated traits when predicting expensive and labor-intensive quality parameters.

**Abstract:**

Multi-trait genomic prediction models can be used to predict labor-intensive or expensive correlated traits where phenotyping depth of correlated traits could be larger than phenotyping depth of targeted traits, reducing resources and improving prediction accuracy. This is particularly important in the context of allocating phenotyping resource in plant breeding programs. The objective of this work was to evaluate multi-trait models predictive ability with different depth of phenotypic information from correlated traits. We evaluated 495 wheat advanced breeding lines for eight baking quality traits which were genotyped with genotyping-by-sequencing. Through different approaches for cross-validation, we evaluated the predictive ability of a single-trait model and a multi-trait model. Moreover, we evaluated different sizes of the training population (from 50 to 396 individuals) for the trait of interest, different depth of phenotypic information for correlated traits (50 and 100%) and the number of correlated traits to be used (one to three). There was no loss in the predictive ability by reducing the training population up to a 30% (149 individuals) when using correlated traits. A multi-trait model with one highly correlated trait phenotyped for both the training and testing sets was the best model considering phenotyping resources and the gain in predictive ability. The inclusion of correlated traits in the training and testing lines is a strategic approach to replace phenotyping of labor-intensive and high cost traits in a breeding program.

**Electronic supplementary material:**

The online version of this article (10.1007/s00122-018-3186-3) contains supplementary material, which is available to authorized users.

## Introduction

Wheat is one of the most important staple food crops of humans (Shewry and Hey 2015), providing 18% of the total caloric intake of the world (FAO 2017). Bread is one of the most important end-use products of wheat; therefore, improving bread quality is a key aspect of wheat improvement. Baking bread quality is a complex trait with quantitative inheritance, derived by several individual traits each one with a different level of environmental influence (Williams et al. 2008). One of the traits that determines baking quality is gluten strength, which is a key factor for loaf volume that determines rising and shape maintenance during the baking process (MacRitchie 1992). Gluten strength is affected by the proportion of glutenins and gliadins polypeptides (75–80% of total proteins) synthetized by each wheat variety (MacRitchie 1992). Therefore, the amount and quality of proteins determine gluten strength and can be evaluated with the sedimentation volume value (MacRitchie 1992). Protein quantity also determines the stability of the dough to create a network and retain water, which is called wet gluten (WG). There is a complex interaction between proteins and other components such as pentosans (Hamer et al. 2009) making dough strength predictability from chemical composition very difficult. Therefore, rheological tests are required. The alveograph is used to investigate the stretching properties of the dough. The total energy required for breaking a standardized bubble is called baking strength (*W*). The length of the curve (*L*), or the time required to break it, is the extensibility, and the height of the peak (*P*) represents the tenacity or maximum dough resistance to rupture (Indrani et al. 2007). All these traits are used to select varieties with better bread quality (Vázquez 2009).

Baking quality is a complex quantitative trait, and several breeding strategies have been successfully used to improve complex traits. In the pre-genomic era, common breeding strategies involved the use of classical quantitative genetic approaches (Lynch and Walsh 1998), including pedigree information to estimate best linear unbiased predictors (BLUPs; Henderson and Quaas 1976). With the widespread availability of molecular markers, genomic approaches have been used (Lande and Thompson 1990). One of the most widely used strategies involves using the additive relationship matrix estimated from markers instead of the additive relationship matrix estimated from pedigree with BLUP models. This was the beginning of the genomic selection (GS) era, and the new BLUP model was called a G-BLUP model (Meuwissen et al. 2001; Habier et al. 2007). The G-BLUP model is equivalent to a ridge regression BLUP model (RR-BLUP; Habier et al. 2007) and to Bayesian models that assume Gaussian priors for marker effects (i.e., Bayesian ridge regression; VanRaden 2008). The latter models compute genetic breeding values (GEBV) adding up all marker effects which were estimated assuming a normal distribution of marker effects with common variance. These models have been the model of choice in most of the prediction scenarios due to their high predictive ability and simple implementation (Heslot et al. 2015). However, other models that assume heterogeneity of variances and different distributions for marker effects performing variable selection (de los Campos et al. 2013) might have higher predictive ability in specific situations (reviewed in Lorenz et al. 2011).

Classical quantitative genetics theory also provides the necessary framework for selecting multiple traits, and classical approaches used for multi-trait selection include an independent culling approach, tandem and index selection (Falconer and Mackay 1996). Multi-trait (MT) selection is justified only when traits are genetically correlated (Henderson and Quaas 1976). This correlation can be the result of pleiotropy or linkage disequilibrium between genes (Falconer and Mackay 1996). A particular case of selection indices [i.e., Smith–Hazel Index (Smith 1936; Hazel 1943)] uses the correlation and the phenotypic and genotypic variance–covariance matrices among traits to estimate the net merit of genotypes. Multi-trait predictions have been extended for the use of genomic information (Calus and Veerkamp 2011; Ceron-Rojas et al. 2015). In this case, the correlation among observations is a function of the additive genetic correlation among traits and the additive genetic relationship among individuals (Calus and Veerkamp 2011). This same strategy was applied to predict lines in the context of genotype by environment interaction using the environmental correlation as multi-trait (Burgueño et al. 2012). Similar to single-trait models, multi-trait models could assume different marker distributions to estimate the breeding values. Calus and Veerkamp (2011) presented three models: one model estimates all marker effects assuming a unique variance (multi-trait G-BLUP); the other two models perform variable selection; one assumes the same variance for all the SNPs (BayesCπ), while the other assumes different variances whether the SNPs were or not associated with a quantitative trait loci (QTL; BayesSSVS). In this study, the differences in performance among methods were small (Calus and Veerkamp 2011). Jia and Jannink (2012) compared three models that were similar to those of Calus and Veerkamp (2011), but used different genetic architectures for the traits, and found that the models assuming different variances (BayesA) and performing variable selection (BayesCπ) were best when QTL of major effects were simulated. However, for truly quantitative traits, models assuming normal distribution of marker effects and unique variances were similar to more complex models. Therefore, a simple model using additive relationship matrix as variance–covariance matrix among individuals seems to perform well for predicting single- (Habier et al. 2007) and multi-trait models (Jia and Jannink 2012; Guo et al. 2014).

Prediction of new un-phenotyped individuals has been studied using multi-trait and single-trait genomic predictions models (Calus and Veerkamp 2011; Jia and Jannink 2012; Guo et al. 2014). Calus and Veerkamp (2011) did not find significant differences between both models using simulated data for predicting traits with different but high heritability (*h*^2^ = 0.6 and *h*^2^ = 0.9). However, the multi-trait model showed good performance to predict low heritability traits with the help of correlated traits with high heritability (Jia and Jannink 2012; Guo et al. 2014) and was optimal when the genetic architecture was explained by major QTL. Finally, the advantage of multi-trait models to predict new un-phenotyped individuals was not so obvious when using experimental data for diseases resistance in pine (Jia and Jannink 2012), grain yield and protein content in rice (Schulthess et al. 2016), several traits in maize (Dos Santos et al. 2016) and grain yield in wheat through normalized difference vegetation index (NDVI) and canopy temperature (Sun et al. 2017). Therefore, the superiority of multi-trait models for predicting un-phenotyped individuals when using simulated data was not confirmed using experimental data.

On the other hand, simulated and empirical studies show that multi-trait models were useful for predicting traits when individuals were partially phenotyped (Rutkoski et al. 2012; Jia and Jannink 2012; Guo et al. 2014; Rutkoski et al. 2016; Hayes et al. 2017; Sun et al. 2017). Both Rutkoski et al. 2012 and Sun et al. (2017) found advantages of multi-trait models using correlated traits from high-throughput phenotyping (i.e., NDVI and canopy temperature) in wheat. Jia and Jannink (2012) also found an improvement in predicting rust gall volume and the presence or absence of rust in pine. Finally, Hayes et al. 2017 found that end-use quality traits could be better predicted using near-infrared (NIR) or nuclear magnetic resonance (NMR). The results show that multi-trait models could be used to decide the optimal depth of phenotyping for each trait, mainly for expensive or difficult-to-measure traits (Guo et al. 2014). However, there is no evaluation on how much phenotyping of the labor-intensive and expensive traits could be replaced by evaluating correlated inexpensive traits.

The objective of this work was to evaluate how multi-trait models could be used to optimize phenotyping resource allocation in breeding programs for expensive or labor-intensive traits. Specifically, we evaluated whether the phenotyping of expensive and labor-intensive traits could be replaced by the use of simple-to-measure traits without affecting the predictive ability; and we compared phenotyping strategies including the use of purposefully unbalanced designs that would require phenotyping the same number of individuals for each trait but in an unbalanced manner such that the population evaluated is significantly larger and therefore the predictive ability potentially larger.

## Materials and methods

### Plant material

Advanced inbred lines from the wheat breeding program from the ‘Instituto Nacional de Investigación Agropecuaria’ (INIA), Uruguay, were used for this study. We used 820 advanced inbred lines for the phenotypic analysis and 1974 advanced inbred lines for the genotypic analyses. Finally, only 495 advanced inbreed lines, having both genotypic and phenotypic information, were used to adjust the genomic selection models.

### Phenotyping

The advanced inbred lines from INIA’s wheat breeding program were phenotyped for eight baking quality traits. The lines were grown in 82 trials in nine location-year combinations (environment) as part of the wheat breeding program (Table S1). There were trials from different breeding scheme stages in each environment: preliminary and advanced trials, and two maturity groups: short and long maturity. The traits were evaluated in field nurseries located in La Estanzuela (34°20′S, 57°42′W; 81 m asl), Colonia, Uruguay, over 5 years (2010–2014). Additionally, approximately one-third of the lines were also evaluated in Young (32°76′S, 57°57′W; 85 m asl) and Ruta2 (33°45′S, 57°90′W; 95 m asl; Table S1). There were two to eight lines that linked the trials within and among environments (Table S2).

Eight baking quality traits were evaluated in 820 experimental advanced inbred lines. Most lines were evaluated in a single environment (~ 680 lines), while the most promissory lines were evaluated in multiple environments (~ 140 lines). Grain protein content (Pt) and test weight (TW) were determined with methods 46–12 and 55–10 of the American Association of Cereal Chemists (AACC 2000), respectively. Refined flour was obtained using the Bühler Mill (AACC Approved Method 26–21A, AACC 2000) method or equivalent. Flour attributes: wet gluten content (WG), alveograph parameters (*W* and *L*), and mixograph parameters (stability (MH) and time (MT)), were evaluated using the AACC methods 38–12, 54–30 and 54–40 (AACC 2000), respectively. Sedimentation volume (SV) was measured according to Peña et al. (1990).

### Phenotypic analyses

Best linear unbiased estimation (BLUE) for bread baking quality traits was estimated from data coming from the different trials and environments using the following model:

1 $$y_{ijk} = \mu + g_{i} + e_{j} + t_{k\left( j \right)} + \varepsilon_{ijk}$$

where $y_{ijk}$ is the phenotypic value of the *i*-th genotype in *j*-th year location for *k*-th trial, *µ* is the overall mean, *g*_*i*_ is the fixed effect of the *i*-th genotype, *e*_*j*_ is the fixed effect of *j*-th environment, *t*_*k*(*j*)_ is the random effect of the *k*-th trial nested within *j*-th environment, and *ε*_*ijk*_ is the residual error for the *i*-th genotype in the in *j*-th environment and *k*-th trial, where *t*_*k*(*j*)_ and *ε*_*ijk*_ were random variables being *t*_*k*(*j*)_ ~ *N*(0,*σ*_*t*_^2^) and *ε*_*ijk*_ ~ *N*(0, *σ*_*e*_^2^). The BLUEs were estimated using ‘nlme’ (Pinheiro and Bates 2017) and ‘lsmeans’ (Lenth 2016) packages from the R statistical software (R Development Core Team 2016).

To evaluate the genotype by environment interaction and the impact of unbalanced designs, Pearson’s correlation between environments was estimated using BLUEs computed by environment (as in model 1 but without the environmental effect). Additionally, genotypic, environmental and genotype by environment interaction variance components were estimated. Finally, Pearson’s correlation and principal component analysis between traits were estimated to evaluate the correlation among traits using the BLUEs estimated from model 1. The final additive variance–covariance matrix between traits was later estimated from the genomic prediction model.

Broad sense heritability for baking quality traits was estimated as follows (Piepho and Möhring 2007):

2 $$H^{2} = \frac{{\sigma_{\text{g}}^{2} }}{{\sigma_{\text{g}}^{2} + {\raise0.7ex\hbox{${\bar{\nu }}$} \!\mathord{\left/ {\vphantom {{\bar{\nu }} 2}}\right.\kern-0pt} \!\lower0.7ex\hbox{$2$}}}}$$

where *σ*_*g*_^2^ is the genotypic variance and $\bar{\nu }$ the mean variance of the difference of two adjusted means.

### Genotyping

Leaf tissue from 1974 lines was collected, and the CTAB method (Saghai-Maroof et al. 1984) was used to isolate DNA for genotyping-by-sequencing (Poland et al. 2012a). The TASSEL-GBS pipeline (Glaubitz et al. 2014) was run with modifications for non-reference genomes (Poland et al. 2012b). SNPs were filtered setting maximum missing value of 20%. Individuals with more than 50% missing information were also discarded. The initial calling of alleles was conducted on the large number of individuals, and then, marker information for the 495 individuals for which phenotypic information was available was used. Therefore, the remaining 6655 SNPs were those with minor allele frequency larger than 0.05 across the 495 individuals. SNP imputation was conducted using the multivariate normal expectation maximization method (Endelman 2011; Poland et al. 2012a). The additive relationship matrix (*K*) was estimated as $K = \frac{WW'}{{2\sum p_{m} q_{m} }}$ where *W* is the centered genotypic matrix, $W_{im} = X_{im} + \left( {1 - 2p_{m} } \right)$ with $X_{im}$ the genotype of the *i*-th individual for the m-th marker as {− 1,0,1} and $p_{m} , q_{m}$ the allelic frequencies where $q_{m} = 1 - p_{m}$ (Endelman and Jannink 2012). *K* was estimated using the ‘rrBLUP’ package in R Statistical Software (Endelman 2011).

### Genomic prediction models

Predictions were obtained from 495 inbreed lines with phenotypic and genotypic information.

#### Single-trait model

**Fig. 1:**
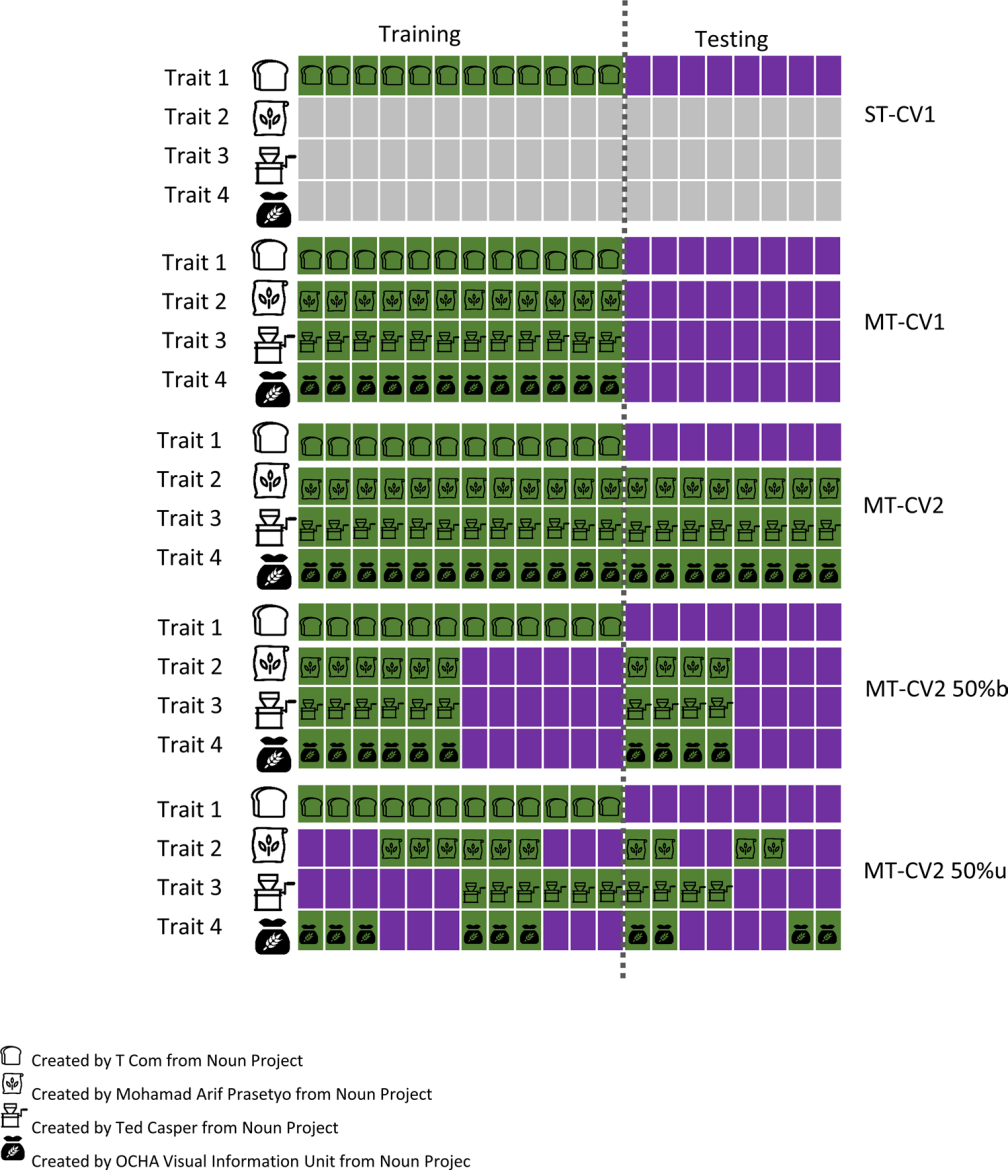
Predictive strategies for an expensive or difficult-to-measure trait (i.e., Trait 1). ST-CV1: single-trait prediction cross-validation 1 where a trait is predicted at a time; we used 60% of individuals as the training population (phenotyped and genotyped, green) and 40% of the individuals as the testing population (genotyped but not phenotyped, purple) as an example; MT-CV1: multi-trait prediction for new un-phenotyped individuals; we used 60% of individuals as the training population (phenotyped for all traits and genotyped, green) and 40% of the individuals as the testing population (genotyped but not phenotyped for any trait, purple) as an example; MT-CV2: multi-trait prediction with cross-validation 2 where 100% of the information from three correlated traits is available for the individuals to be predicted; we used 60% of individuals as the training population (phenotyped for all traits and genotyped, green) and 40% of the individuals as the testing population (phenotyped for correlated traits but not for the targeted trait, and genotyped, purple) as an example; MT-CV2-50%b: multi-trait prediction cross-validation 2 with 50% of balanced information in three correlated traits where the same 50% of individuals were phenotyped for each correlated traits; MT-CV2-50%u: multi-trait prediction cross-validation 2 with 50% of unbalanced information in three correlated traits where 50% of individuals were phenotyped for each correlated trait but in an unbalanced manner such that each individual is not necessarily phenotyped for all correlated traits. Rectangles represent lines and colors the presence (green) or absence (purple) of phenotypic information (color figure online)

Using the single-trait model (ST), the prediction performance values were obtained for the eight baking quality traits using a Bayesian ridge regression (BRR) model by trait.

3 $$y_{i} = 1\mu + \mathop \sum \limits_{j = 1}^{j = p} x_{ij} \beta_{j} + \varepsilon_{i}$$

where $y_{i}$ is the adjusted phenotypic mean of individual *i* for a single trait; *μ* is the overall mean; $x_{ij}$ the score of the *j*-th SNP in individual *i*; $\beta_{j}$ is the effect of *j*-th marker; and *ε*_*i*_ is the vector of residual errors. The conditional prior distribution is $\varepsilon_{i} \,\sim\,N\left( {0, \sigma^{2} } \right)$ with $\sigma^{2} \,\sim\,\chi^{ - 2} (\sigma^{2} |{\text{d}}f,S$) for residuals, and $\beta_{j} \,\sim\,N\left( {0,\sigma_{\beta }^{2} } \right)$ with $\sigma_{\beta }^{2} \,\sim\,\chi^{ - 2} ({\text{d}}f_{\beta } ,S_{\beta }$) for genotypic values. Genotypic effects (*g*_*i*_) were predicted as $g_{i} = \sum\nolimits_{j = 1}^{j = p} {x_{ij} \hat{\beta }_{j} }$. Starting values were set for the degrees of freedom of the inverse Chi-squared distributions (d*f*) as 5 and scale parameters were calculated as *S* = var(*y*) × 0.5 following Pérez and de los Campos (2014). We used 1500 burn-in and 3000 iterations for the Gibbs sampler algorithm implemented in ‘BGLR’ package (Pérez and de los Campos 2014). Prediction accuracies for the ST model were estimated using only one cross-validation approach CV1 (de Leon et al. 2016), explained below and in Fig. 1.

#### Multi-trait models

Multi-trait models (MT) were estimated fitting a Bayesian multivariate Gaussian model estimating an unstructured variance–covariance matrix between traits (∑) and a residual matrix (*R*). The multi-trait model is:

4 $$y = 1\mu + Zu + \varepsilon$$

where *y* is a vector of *N *× *t* length (*N* individuals and *t* traits), *µ* is the means vector of length *N *× *t*; *u* is a vector of predicted genetic values of the individuals for all traits with $u\,\sim\,N\left( {0, \sum \otimes K} \right)$ and *ε* is a vector of residuals with $\varepsilon \,\sim\,N\left( {0,R \otimes I} \right)$, where *K* is the realized additive relationship matrix among individuals estimated from the markers, and ∑ and *R* are the variance–covariance matrices for the genetic and residual effects for each individual in all traits, respectively, estimated using a Gibbs sampler algorithm with 1500 burn-in and 3000 iterations. ∑ was estimated as an unstructured matrix and *R* as a diagonal matrix. We used a diagonal matrix for *R* instead of an unstructured matrix because the predictive ability was higher with the diagonal matrix. To estimate ∑ and *R*, scaled inverse Chi-square prior distributions were assigned to ∑ ~ $\chi^{ - 2} \left( {{\text{d}}f_{\varSigma } ,S_{\varSigma } } \right)$ and *R* ~ $\chi^{ - 2} \left( {{\text{d}}f_{\text{R}} ,S_{\text{R}} } \right)$, with arbitrary assigned initial scale random matrix $S_{\varSigma }$ = I_t_, $S_{R} = 1_{t}$ and degrees of freedom ${\text{d}}f_{\varSigma }$ = 4 and ${\text{d}}f_{R} = 1_{t}$. The predictions were obtained using the ‘MTM’ package in *R* (de los Campos and Grüneberg 2016). Finally, a multi-trait model without marker information was evaluated using the same multi-trait model but with an identity matrix for *K* instead of the realized additive relationship matrix.

### Cross-validation scheme

Prediction accuracies were estimated using two main strategies of cross-validation also shown in Fig. 1. The first cross-validation strategy (CV1 following de Leon et al. 2016) used phenotypic and genotypic information from a random set of the advanced inbred lines to train the model (for example, 60% of the population or 297 individuals). Then, the remaining lines (for example, 40% or 198) were predicted using genotypic data only. Pearson’s correlations between the adjusted phenotypic means (model 1) and their predicted values (model 3) were estimated. This process was iterated 100 times selecting different sets of lines each time. This scheme of cross-validation was used for ST and MT models (ST-CV1 and MT-CV1).

The second cross-validation strategy (CV2 following de Leon et al. 2016) used phenotypic and genotypic information from a random set of lines (for example 60% or 297 individuals) from the trait of interest to train the model. In addition, phenotypic and genotypic information for all lines of correlated traits was used. The trait of interested was predicted in the lines not phenotyped for the trait of interest (for example, 40% or 198 individuals, Fig. 1). Pearson’s correlations between adjusted phenotypic means (model 1) and predicted values (model 4) were estimated. This process was iterated 100 times selecting different set of lines each time. This scheme of cross-validation was used only for the MT models (MT-CV2). Multi-trait models were used to predict traits including information from up to three correlated traits which were chosen based on their relationships revealed by principal component analyses and their Pearson’s correlations.

### Improving efficiency of phenotyping

In order to evaluate the possibility to train the models with fewer phenotyped lines, we trained the MT models using 50 to 396 individuals (10 to 80%) from the training population and 100% of the lines phenotyped for the other correlated traits for *W*, *L* and MH. This strategy was used to predict each trait at a time using three correlated traits from the same group of traits (4*T*), two traits from the group (3*T*) or one trait from the group (2*T*). The 2*T* models were constructed with SV for both *W* and MH and with WG for *L*. The 3*T* models were constructed with SV and MH for *W*, SV and TW for MH, and WG and Pt for *L*. We evaluated the accuracy of the predictions using 100 iterations in all traits and CV2.

#### Prediction for alveograph and mixograph parameters (MH, *W* and *L*)

In order to improve the efficiency to predict complex traits such as the parameters from the alveograph or mixograph, we compared the prediction of MH, *W* and *L* using different depth of phenotypic information on correlated traits. The mixograph parameter MT was not evaluated because it is poorly correlated with other traits in its group. First, we evaluated different sizes of this training population for the predicted traits when information on the other three traits was present only in 50% of the lines in a balanced or unbalanced manner. We used the same approach as before with 10–80% of the training population for predicting the traits but using phenotypic information for only 50% of the lines for each correlated trait. To mask 50% of the lines, we followed two strategies: designed as balanced (MT-CV2-50%b) or unbalanced (MT-CV2-50%u). The balanced strategy masks the same 50% of the lines in all correlated traits. In the unbalanced strategy, each trait is also phenotyped in 50% of the individuals, but different individuals are phenotyped for different traits. To mask 50% of the lines, both, the training and testing sets were divided in four equal parts. Then, two different sets from the training and two different sets from the testing were masked in each correlated trait. The lines were randomly assigned to each of the four sets. This procedure was conducted for 100 random iterations (Fig. 1).

## Results

### Phenotypic characterization of the population

**Fig. 2:**
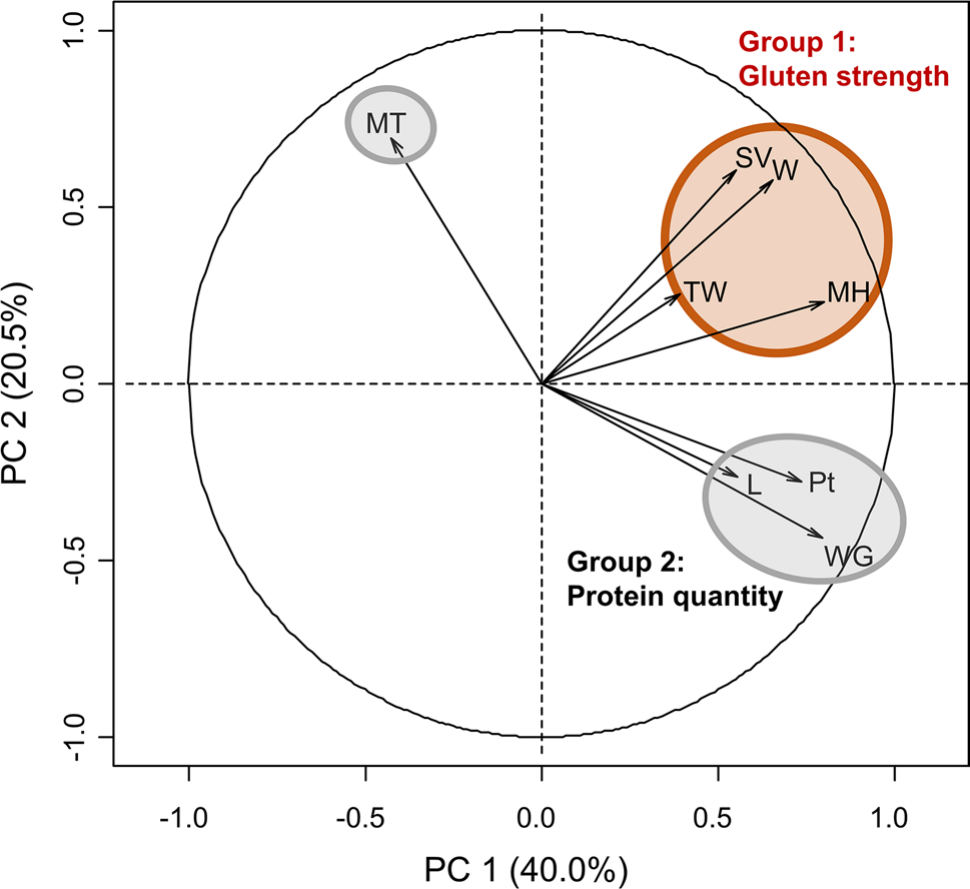
Principal component analysis for the eight baking bread quality traits where the total phenotypic variance explained by each principal component is indicated

**Fig. 3:**
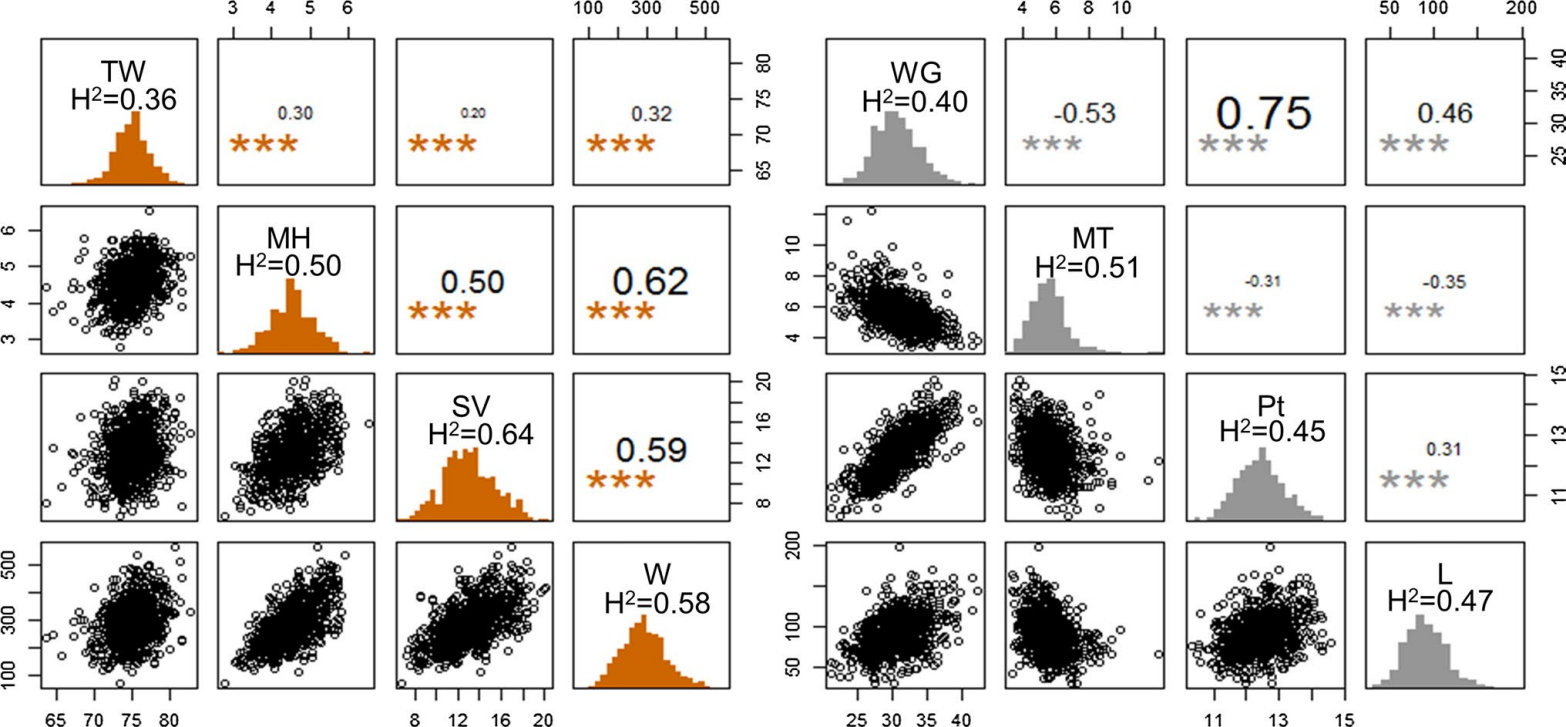
Scatter plot matrix with Pearson’s correlations and phenotypic distributions and trait heritability (on the diagonal) for each group of traits. Group 1 (left) including: TW, test weight; MH, mixograph height; SV, SDS sedimentation volume; *W*, alveograph parameter *W*. Group 2 (right) including: WG, wet gluten; MT, mixing time; Pt, grain protein content; *L*, alveograph *L*. *** indicates significantly different from 0 with *α* = 0.05

Genotypic means for the multi-environment evaluation were used for genomic predictions since genotype by environment interaction among years was low (i.e., high correlation between environments, large heritability across environments, and relatively low proportion of the total phenotypic variance explained by the genotype by environment interaction; Table S2). Two groups of correlated traits were defined using principal component analysis (Fig. 2) and Pearson’s correlation between traits (Fig. 3). The first and second principal components explained 40 and 20% of the total phenotypic variance, respectively. Both groups were represented by four traits. Group 1 includes MH, SV and *W* with high correlation and TW with intermediate correlation with all other traits (Fig. 3). Group 2 includes Pt, WG, MT and *L*. MT had a low and negative correlation with all traits in this group. The heritability for each trait was medium (0.36–0.64, Fig. 3).

### Multi-trait genomic predictions

**Fig. 4:**
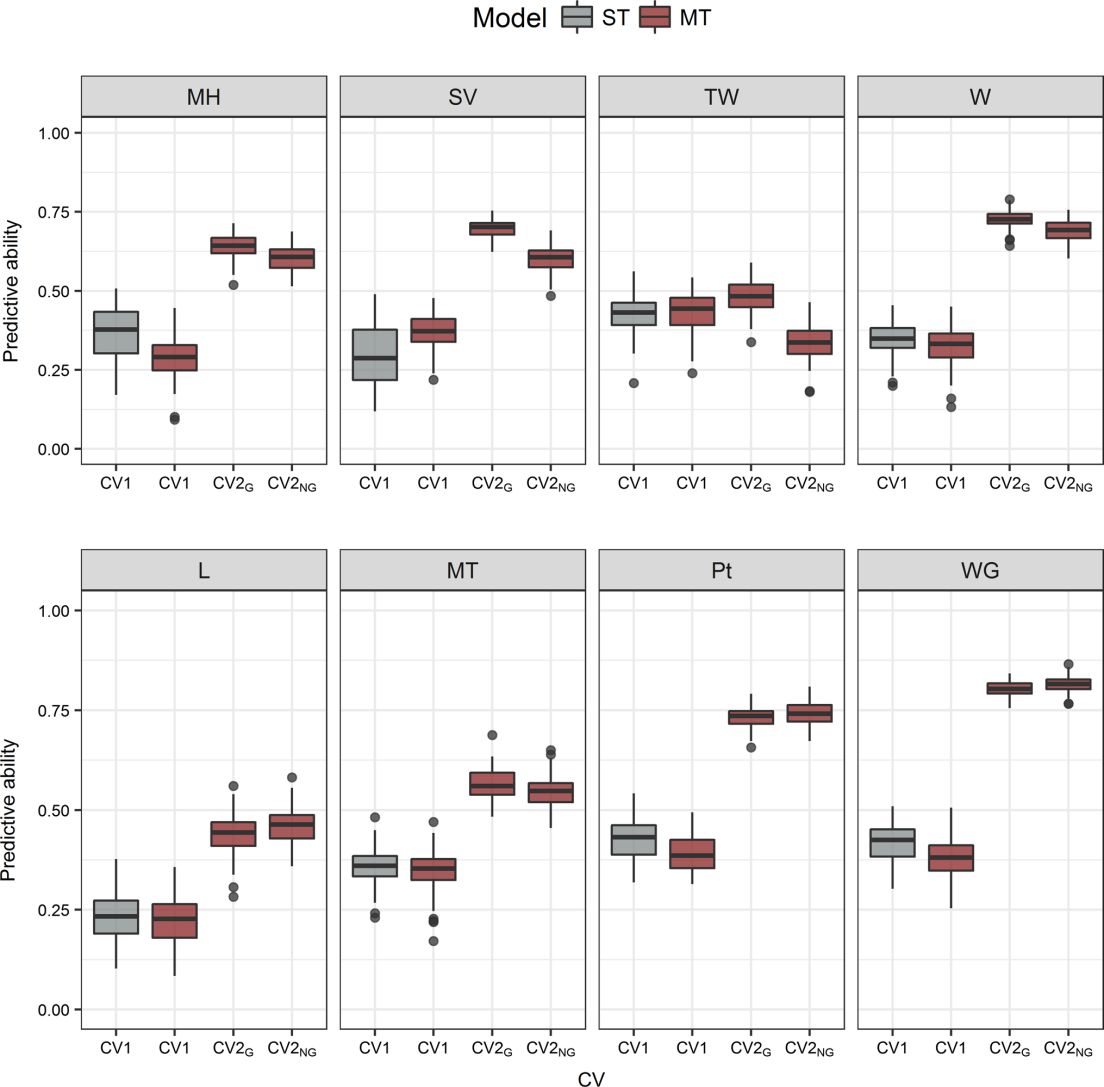
Predictive ability ($r\left( {y,\hat{y}} \right)$) for eight baking bread quality traits. Single-trait prediction model (ST-CV1), and multi-trait prediction model (MT) with two schemes of cross-validation (MT-CV1, predicting new individuals and MT-CV2, predicting individuals phenotyped for correlated traits) with or without genotypic information (CV2_G_ and CV2_NG_, respectively). Group 1 of traits (above): TW, test weight; MH, mixograph height; SV, SDS sedimentation volume; W, alveograph parameter W. Group 2 of traits (below): WG, wet gluten; MT, mixograph parameter-mixing time; Pt, grain protein content; L: alveograph parameter *L*

Similar predictive ability of ST and MT models was found for predicting new un-phenotype individuals (ST-CV1 and MT-CV1, Fig. 4). The traits with the highest predictive ability were TW and Pt ($r_{{\left( {y,\hat{y}} \right)}} = 0.43$ for both), while the trait with the lowest predictive ability was *L* ($r_{{\left( {y,\hat{y}} \right)}} = 0.24$, Fig. 4).

#### Multi-trait predictions using correlated traits

Using information of correlated traits from predicted individuals increased the predictive ability for all traits (MT-CV1 vs MT-CV2, Fig. 4). The improvement in predictive ability through CV2 was different for each trait and related to the correlation between traits. In the group 1, TW was the trait with the smallest increase in predictive ability (Fig. 4) due to its low correlation to other traits (Fig. 3). On the other hand, the increase obtained for highly correlated traits, MT, W and SV, was high. In the group 2, WG was the trait with the largest increase in predictive ability, and this was the trait with the highest correlation with the others three traits in the group (Figs. 3, 4).

#### Replace phenotyping

**Fig. 5:**
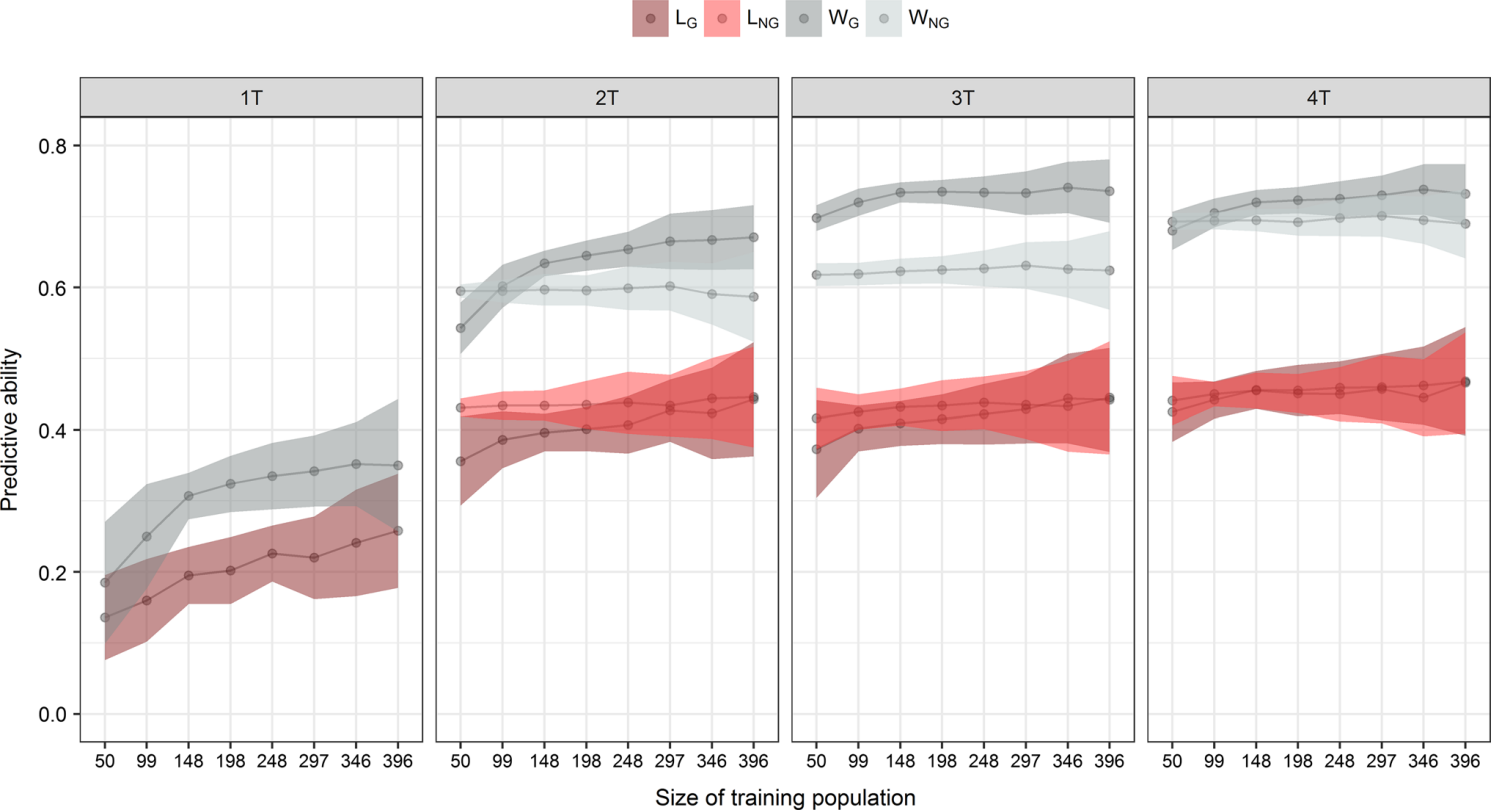
Predictive ability ($r\left( {y,\hat{y}} \right)$) and standard deviation (shadowed interval) for the alveograph parameters W and L using different sizes of the training population (*N* = 495) on the predicted trait. The traits were predicted using the multi-trait model cross-validation 2 (MT-CV2) with four, three or two traits (4*T*, 3*T* and 2*T*), and single-trait model (ST-CV1) to predict one trait (1*T*). Prediction was assessed using genotypic information (*W*_G_ and *L*_G_) and without genotypic information (*W*_NG_ and *L*_NG_)

Using correlated traits from predicted individuals, the training population size can be reduced up to 30% of its size without significantly affecting the predictive ability of the model (Fig. 5). In addition, the inclusion of marker information improved 2–14% the predictive ability of multi-trait models for *W* and there was no improvement for *L* trait (Fig. 5).

One highly correlated trait (2*T*) increased more than 50% the predictive ability compared to the single-trait model for both *W* and *L* (Fig. 5). Two highly correlated traits (3*T*) increased the predictive ability a 14% for W and a 3% for L compared to the model with one correlated trait (Fig. 5). TW did not contribute substantially to *W* predictions, while MH and SV improved the predictive ability regardless of the model used (Fig. 5). In addition, MT and Pt did not contribute to L predictions, while WG increased the predictive ability for *L*.

**Fig. 6:**
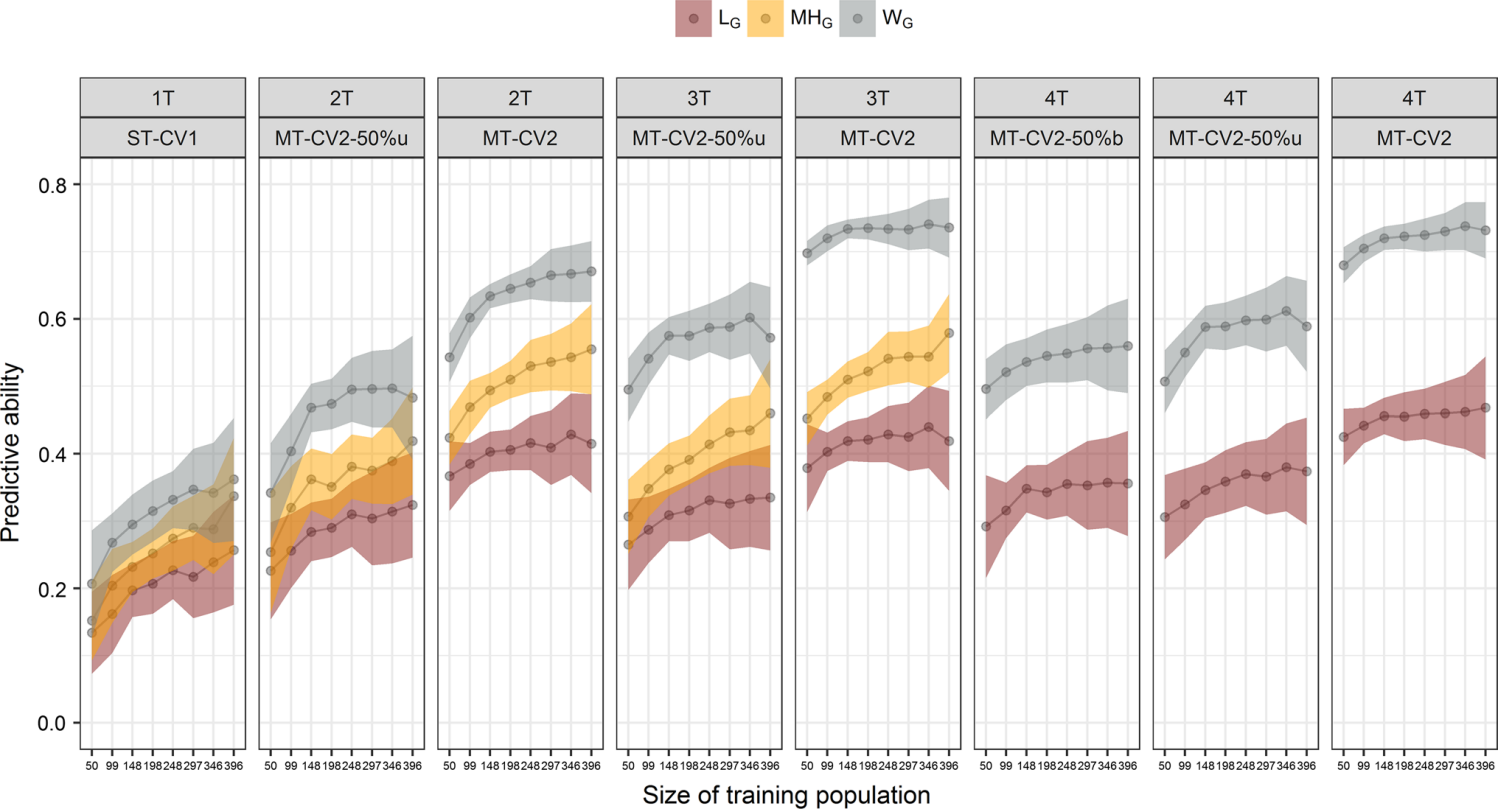
Predictive ability ($r\left( {y,\hat{y}} \right)$) and standard deviation (shadowed interval) of alveograph parameters (*W*, *L*) and mixograph parameter (MH) predictions using different sizes of training population (*N* = 495). Single-trait predictions (ST-CV1) were obtained using the predicted trait to train model (1*T*), or one, two or three correlated traits to train the model (MT-CV2 for 2*T*, 3*T* and 4*T*, respectively). MT-CV2: multi-trait predictions with 100% of information in three correlated traits; MT-CV2-50%u: multi-trait predictions with 50% of unbalanced information in three correlated traits; MT-CV2-50%b: multi-trait predictions with 50% of balanced information in three correlated traits

To predict an expensive trait using correlated traits with equal phenotyping cost, a purposefully unbalanced phenotyping design with 50% of each trait was explored (MT-CV2 50%u). In this case, each trait was phenotyped for 50% of the individuals but for different individuals with some overlaps. This strategy yielded higher predictive ability than using even more phenotyping but only in the training population (MT-CV1 vs MT-CV2 50%u, Fig. 6). For example, 396 individuals phenotyped for *W* on a ST model had a predictive ability of 0.361 ± 0.09, while phenotyping 99 individuals for *W* and 495 for SV had a predictive ability of 0.404 ± 0.06 (Fig. 6). The predictive ability using MT-CV2 50%u for two traits was between 28 and 34% higher than the ST model for MH, *W* and *L* (Fig. 6). Deep phenotyping on correlated trait always reached higher predictive ability than reducing the phenotyping to 50% of the lines for these traits. The predictions obtained using an unbalanced strategy were slightly larger than using the balanced strategy. However, there were no differences in the predictive ability of both strategies used to phenotype 50% of the lines.

## Discussion

Our grouping of traits, where one group was associated with gluten strength and the other one was related to protein quantity, is similar to that found in Vázquez et al. (2012). The high correlation we found between MH, *W* and SV was also found in others studies (Peña et al. 1994; Ruiz and Carillo 1995; Indrani et al. 2007). Model predictive ability using single-trait prediction through CV1 with 40% of randomly masked individuals (198 individuals) was lower (between 0.24 and 0.43) than previously found in Battenfield et al. 2016 (between 0.45 and 0.60) predicted using 20% randomly masked individuals. In addition, they were higher than those found in Hayes et al. (2017) although the strategy used to predict performance was different; here, we used cross-validation approaches and Hayes et al. (2017) predicted traits across years and locations.

### Multi-trait genomic predictions

Predicting new un-phenotyped individuals is always a challenge, and different strategies have been used to improve the predictive ability in those circumstances. The use of correlated trait responses has been effective when the predicted trait is of low heritability and the highly correlated trait is of high heritability (Jia and Jannink 2012; Guo et al. 2014; Jiang et al. 2015). These has been thoroughly studied both theoretically and empirically, within classic quantitative genetic studies (Falconer and Mackay 1996; Lynch and Walsh 1998) and with genomic studies (Calus and Veerkamp 2011; Jia and Jannink 2012; Guo et al. 2014; Jiang et al. 2015). However, for very complex polygenic traits, there is a small advantage of a multi-trait model with correlated responses even with high heritability differences among traits (Jia and Jannink 2012). Furthermore, studies with real experimental data from quantitative genetics using genomic information did not show a significant improvement of multi-trait models in mice (Jiang et al. 2015), avocado (He et al. 2016), maize (Dos Santos et al. 2016) or rice (Schulthess et al. 2016). We found a similar response, where the multi-trait model (MT-CV1) did not perform better than the single-trait model (ST-CV1). This was somewhat expected because although our traits were correlated, all traits have high heritability and because of the theoretical complexity of the traits (Nelson et al. 2006; Sun et al. 2008; Li et al. 2016).

#### Predictions for partially phenotyped individuals

Correlated traits can also be used to predict a correlated response when the individuals have been phenotyped for other traits (Rutkoski et al. 2012; Jia and Jannink 2012). Some previous work showed high prediction accuracy using highly correlated traits, but not with intermediate to low correlated traits (Calus and Veerkamp 2011; Jia and Jannink 2012; Jiang et al. 2015). We found the same trend in our study, where the use of correlated responses using information from other traits increased the predictive ability of models, and the predictive ability was directly related to the correlation between traits. Therefore, correlated traits in the lines to be predicted can be used to increase the predictive ability of the models.

#### Predictions for replace phenotyping

We showed that the use of correlated traits from predicted individuals (MT-CV2) increase the predictive ability of the models, and this was somewhat already shown. The next question we wanted to address was how much could we reduce the depth of phenotyping of an expensive trait (i.e., *W* or *L* in or study based on prices from the Canadian Grain Commission, Wheat Marketing Center, and AIB International), and in consequence the training population size, by using correlated traits without compromising the predictive ability of the model. It has been widely proven that smaller population sizes reduce prediction accuracy (Asoro et al. 2011; Heffner et al. 2011; Rincent et al. 2012; Akdemir et al. 2015; Rutkoski et al. 2015; Cericola et al. 2017). However, our hypothesis was that by using correlated traits we could somewhat offset the effect of smaller population sizes. This hypothesis was tested with a range of population sizes (i.e., 50 to 396 individuals or 10 to 80%) and with real data. We found that the training population could be reduced up to 30% of the total population without significantly affecting the predictive ability of the models if correlated traits were used. Our results show that it is possible to effectively design training populations where expensive or difficult-to-phenotype traits are phenotyped at a smaller depth than cheaper or easier-to-phenotype correlated traits. Our results were obtained with individuals from the training population chosen at random. Other studies (Akdemir et al. 2015; Isidro et al. 2015; Rincent et al. 2017) found that optimizing the training population to select the most predictive individuals instead of using a random sample increases the predictive ability. We would therefore expect that our results are the baseline for the gain that could be achieved by using replaced phenotyping when the training population is optimized.

We found that the increase in predictive ability including marker information was marginal compared to the multi-trait model using phenotypic data from correlated traits when a large number of highly correlated traits were used. However, Crain et al. (2018) showed the importance of using marker information to predict traits in a new environment where phenotypic correlation could be lower than expected due to genotype by environment interaction. In our work, we conducted cross-validation using the same population, but this will not be the situation during selection in a breeding program.

We showed that expensive traits could be assessed for fewer individuals without affecting its predictive ability if information of correlated trait is used from all individuals. This requires extensive phenotyping for all correlated traits. Our results showed that the predictive ability using 50% of correlated information (MT-CV2-50%u) was lower than in the full phenotyping (MT-CV2) models. However, the predictive ability was still high. In addition, the use of an unbalanced strategy to reduce phenotyping on correlated traits was slightly better than reducing phenotyping using a balanced strategy (MT-CV2-50%u vs MT-CV2-50%b). Therefore, unbalanced phenotyping of correlated traits could be another approach to predict traits that are expensive or labor-intensive.

Finally, we evaluated whether the inclusion of more than one trait increases the predictive ability of the model if this trait is highly correlated. We found that models with two highly correlated traits are better than models with one highly correlated trait. However, the increase in predictions is low with the addition of a second correlated trait. Therefore, it will be important to evaluate the cost of prediction using two instead of one correlated trait, balancing gain in accuracy with the costs of using another trait to help predictions. The use of mildly correlated traits such as TW, MT, and Pt was not useful.

## Conclusion

The use of multi-trait models is useful to improve the predictive ability of partially phenotyped individuals. Expensive or difficult-to-phenotype traits can be phenotyped in smaller population sizes if the predicted individuals are phenotyped fully or partially for less expensive correlated traits. Particularly, we found that the use of only one correlated trait in the model was the most effective way to increase the predictive ability with fewer resources.

### Author contribution statement

DV and MQ designed the phenotyping experiments. BL and PS performed genotyping analyses. BL, LG and IA performed statistical analyses. BL, LG, PS and IA wrote the paper. LG designed the study and hypothesis. All authors read and approved the final manuscript.

## Electronic supplementary material

Below is the link to the electronic supplementary material.

Supplementary material 1 (PDF 232 kb)
