# Supplementary material for: Resource allocation optimization with multi-trait genomic prediction for bread wheat (Triticum aestivum L.) baking quality
Source: Theor Appl Genet. 2018 Sep 19;131(12):2719–31. doi: 10.1007/s00122-018-3186-3 (PMC6244535; doi:10.1007/s00122-018-3186-3)
Supplement: Supplementary file 1 — Supplementary material 1 (PDF 232 kb) [file 122_2018_3186_MOESM1_ESM.pdf]

Table S1. Number of trials and lines present in each environment (location-year combination).

	La Estanzuela					Ruta2	Young		
	2010	2011	2012	2013	2014	2013	2011	2012	2014
**#Trials**	11	15	15	14	15	3	3	3	3
**#Lines**	165	170	226	218	207	51	42	44	50

Table S2. Pearson‘s correlations and standard error, general mean, genetic variance, environmental variance and genotype by environment interaction variance and broad sense heritability for eight quality traits between consecutive years in La Estanzuela experimental station, Uruguay.

	**TW**	**MH**	**SV**	**W**	**WG**	**MT**	**Pt**	**L**
$r_{2010\text{-}2011}$ (n=32)	0.65±0.14	0.19±0.18	0.46±0.16	0.43±0.20	0.61±0.14	0.60±0.15	0.74±0.12	0.62±0.17
$r_{2011\text{-}2012}$ (n=39)	0.26±0.16	0.37±0.15	0.18±0.16	0.36±0.15	0.31±0.16	0.17±0.16	0.29±0.16	0.18±0.16
$r_{2012\text{-}2013}$ (n=48)	0.33±0.17	0.43±0.13	0.82±0.08	0.53±0.12	0.65±0.11	0.38±0.14	0.39±0.14	0.44±0.13
$r_{2013\text{-}2014}$ (n=46)	0.59±0.12	0.52±0.13	0.66±0.11	0.26±0.15	0.70±0.11	0.38±0.14	0.59±0.12	0.56±0.13
$r_{2011LE\text{-}Y}$ (n=42)	0.68±0.12	0.40±0.14	0.67±0.12	0.57±0.13	0.41±0.14	0.56±0.13	0.32±0.15	0.80±0.10
$r_{2012LE\text{-}Y}$ (n=44)	0.68±0.11	0.58±0.13	0.46±0.14	0.64±0.12	0.79±0.09	0.73±0.11	0.80±0.09	0.34±0.15
$r_{2013LE\text{-}R2}$ (n=51)	0.63±0.13	0.58±0.12	0.60±0.11	0.54±0.12	0.68±0.10	0.32±0.14	0.76±0.09	0.40±0.13
$r_{2014LE\text{-}Y}$ (n=50)	0.46±0.13	0.44±0.13	0.59±0.12	0.59±0.12	0.66±0.11	0.40±0.14	0.72±0.10	0.43±0.13
Mean	75.0	4.56	13.1	290	30.8	12.4	5.64	91.4
$\boldsymbol{\sigma_G^2}$	1.95	0.14	3.39	322	5.22	0.52	0.21	240
$\boldsymbol{\sigma_E^2}$	14.79	0.06	3.50	230	4.67	0.11	0.33	448
$\boldsymbol{\sigma_{GEI}^2}$	3.42	0.14	1.91	236	4.84	0.63	0.29	279
GEI (%)	10	41	22	30	33	50	35	29
H^2	0.36	0.50	0.64	0.58	0.40	0.51	0.45	0.47
